# Improving Attention through Individualized fNIRS Neurofeedback Training: A Pilot Study

**DOI:** 10.3390/brainsci12070862

**Published:** 2022-06-29

**Authors:** Yue Gu, Liu Yang, He Chen, Wenzheng Liu, Zhenhu Liang

**Affiliations:** 1 Key Laboratory of Computer Vision and System (Ministry of Education), School of Computer Science and Engineering, Tianjin University of Technology, Tianjin 300384, China; 18832026962@163.com (L.Y.); 15122258266@163.com (W.L.); 2 Engineering Research Center of Learning-Based Intelligent System (Ministry of Education), Tianjin University of Technology, Tianjin 300384, China; 3 The State Key Laboratory of Cognitive Neuroscience and Learning, Beijing Normal University, Beijing 100875, China; chenhe_bang@163.com; 4 Institute of Electrical Engineering, Yanshan University, Qinhuangdao 066004, China; zhl@ysu.edu.cn; 5 Key Laboratory of Intelligent Rehabilitation and Neuromodulation of Hebei Province, Qinhuangdao 066004, China

**Keywords:** individualized neurofeedback, non-individualized neurofeedback, attention, functional near-infrared spectroscopy

## Abstract

Attention is a particularly important indicator in life, as inattention can lead to many negative consequences. As a non-invasive intervention, real-time neurofeedback training can effectively enhance individuals’ attention adjustment abilities. However, previous studies have neglected to consider differences among individuals. In this study, an individualized neurofeedback training (INT) method based on functional near-infrared spectroscopy (fNIRS) was proposed for attention improvement and compared with non-individualized neurofeedback training (NINT). The neurofeedback channels and thresholds were determined individually for each subject. Then, participants conducted four runs of neurofeedback training. Two attention tests (i.e., AX version of continuous performance task (AX-CPT) and attention network test (ANT)) were used to assess the performance of the neurofeedback training. The length of time that the two groups of participants continuously kept their oxygenated hemoglobin concentration above a threshold showed an increasing trend, and the improvement rate of the INT group was higher than that of the NINT group. The reaction times for both groups showed a downward trend, but the INT group declined more significantly. In the fNIRS data, it was observed that the activation degree of the INT group in the middle and dorsolateral prefrontal areas was higher than that of the NINT group. It is preliminarily proved that the proposed INT method can effectively improve the attention level, and its overall performance is better than that of the NINT method.

## 1. Introduction

Attention refers to the ability of a person to mentally identify and concentrate on something; it is the tendency to selectively process certain stimuli and ignore others. Many human behaviors require high levels of attention and sometimes rely on sustained attention, such as when listening to a lecture or reading a book [1]. Low attention levels or difficulty maintaining attention can bring great harm. For example, attentional deficits are common symptoms of certain neurological disorders such as attention-deficit/hyperactivity disorder. Therefore, it is of immense importance to effectively improve humans’ levels of attention.

With the continuous development of neuroimaging technology, neurofeedback through physiological measurements has become a common means of regulating attention-related neural activities [2]. Neurofeedback is a form of behavioral therapy that is based on the principle of operant conditioning, which trains and conditions the brain through neurofeedback provided by the brain [3]. In this method, participants learn how to use operational conditioning to change their brain activity as needed [4].

At present, the physiological measurements used in neurofeedback are mainly comprised of electroencephalogram (EEG) and functional magnetic resonance imaging (fMRI) technology. EEG signals feature high time resolution and practicality. There have been numerous EEG-based neurofeedback studies. Some research has suggested that real-time EEG neurofeedback can achieve cortical activation and the voluntary adjustment of attention levels [5,6,7]. In addition, fMRI has been found to feature excellent spatial resolution as a neurofeedback measurement tool through real-time data processing [8]. Thus, there have also been numerous studies using fMRI for neurofeedback. Some research has shown that real-time fMRI neurofeedback can be used for single-brain regional brain activation or functional connectivity training between brain regions, ultimately improving the level of attention [9,10,11].

However, although neurofeedback systems using fMRI for physiological measurements have proven to be effective, the relatively high equipment requirements and restriction of participants’ movement continue to be challenging. In order to reduce cost and enhance mobility, functional near-infrared spectroscopy (fNIRS) has emerged in recent years as a novel physiological measurement method and effective alternative. The technology uses oxygenated hemoglobin (Oxy-Hb) and deoxygenated hemoglobin (Deoxy-Hb), which have different absorption effects in near-infrared light. Thus, fNIRS can detect changes in Oxy-Hb and Deoxy-Hb in brain function areas, indirectly reflecting brain activity [12]. Compared with EEG, the preparation needed for fNIRS is minimal. For participants, repeated fNIRS measurements are comparatively more comfortable because there is no need to apply conductive gel. In addition, fNIRS is not susceptible to external electromagnetic interference. Compared with fMRI, fNIRS is lower in cost, more flexible, and more portable [13]. fNIRS has a higher time resolution than fMRI scanners do [14,15]. Moreover, fNIRS is less sensitive to motion artifacts and can adapt to higher degrees of motion [16,17].

Due to the various advantages of fNIRS, it is a better choice for the field of neurofeedback [18]. Thus, in recent years, neurofeedback research using fNIRS as a physiological measurement tool has increased. Marx et al. recruited 12 children with attention deficit and hyperactivity disorder (ADHD) for fNIRS neurofeedback training. After 12 training sessions, the patients had significantly reduced their ADHD symptoms [19]. The study also compared the neurofeedback effects of EEG and fNIRS. The results showed that the fNIRS neurofeedback effect was faster and more effective. Blume et al. used virtual-reality technology to conduct fNIRS neurofeedback training on 90 children with ADHD. After 15 training sessions, the patients’ performance on various ADHD test questionnaires and parent interviews all indicated substantial improvements [20]. Kinoshita and colleagues recruited 24 healthy adult participants for fNIRS neurofeedback training. The target brain area was the frontal cortex. Participants were divided into real and false neurofeedback groups. The results showed that only the real neurofeedback group was tested. The frontal pole region was significantly activated [21]. Hosseini and colleagues recruited 20 healthy adults for neurofeedback training, of which 10 participants received real neurofeedback information and 10 received information that was sham. Compared to the sham neurofeedback group, the executive function performance of the real neurofeedback group was significantly improved [22].

To a certain extent, the above studies confirmed the effect and efficiency of fNIRS neurofeedback, but existing neurofeedback research has not taken into account the differences among individuals. Previously, Grandy et al. proposed the individual alpha frequency for EEG [23], and studies have verified the existence of this difference [24]. At present, most known fNIRS attention neurofeedback target areas are in the prefrontal lobe, but the channels that are mainly activated are not consistent among individuals and the activation levels of the same channels vary. Previous fNIRS neurofeedback has been based on a certain channel or area, and the neurofeedback channel and threshold of each participant was assumed to be the same. Thus, differences among individuals have not been taken into account. Consequently, the present research proposes individualized neurofeedback training (INT).

In summary, in order to improve the effect of fNIRS-based attention neurofeedback training, we propose an INT program. Through pre-experiments, participants’ channels with higher activation level in an attention test task were determined. These channels were used as neurofeedback channels in training. Finally, we calculated the level of blood oxygen concentration that was higher than the threshold during the INT program, as well as the response time change during the attention test task, and compared these with the non-individualized neurofeedback training (NINT) program to evaluate the INT program’s performance.

## 2. Materials and Methods

### 2.1. Participants

A total of 10 college students (all male, aged 24.0 ± 1.8 years old) participated in this experiment. All participants were right-handed, had no known respiratory, cardiovascular, psychiatric, or neurological diseases, and had not undergone any type of neurofeedback training. Participants signed an informed consent form before the experiment and received a small amount of remuneration after completion. This research was approved by the Institutional Review Board of the State Key Laboratory of Cognitive Neuroscience and Learning in Beijing Normal University (CNL_A_0010_010), in accordance with the Declaration of Helsinki.

All participants were randomly divided into two groups: an INT group using individualized channels and thresholds for neurofeedback and an NINT group using common channels and thresholds. Each of the two groups was comprised of five participants.

### 2.2. fNIRS Data Collection

This experiment used the NIRSport function near-infrared imaging equipment (NIRSport, NIRx, Medical Technologies) to detect participants’ brain activity. The equipment uses near-infrared wavelengths of 760 nm and 850 nm. Its working principle is to use the absorption of near-infrared light by different blood components. When the degree is different, the relative concentration changes of Oxy-Hb and Deoxy-Hb are measured. In this experiment, the distance between adjacent light sources and detectors was about 3 cm, and the sampling frequency was 7.8125 Hz.

Since the Oxy-Hb signals exhibit superior sensitivity in task-related signal changes and a greater correlation with blood oxygen level-dependent signals in functional magnetic resonance imaging [6], we used the concentrations of Oxy-Hb as the neurofeedback signals in this study. Meanwhile, for a more comprehensive description of hemodynamic changes, we performed the activation analysis on both Oxy-Hb and Deoxy-Hb signals.

**Figure 1:**
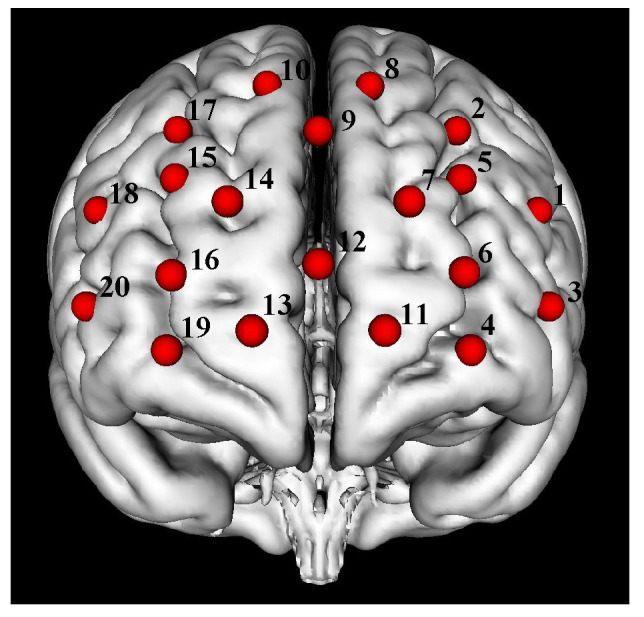
Channel position map. The 20 channels composed of fNIRS optodes were located in the prefrontal area. The red spheres indicate channels and the black numbers show the channel numbers.

Attention is not represented anatomically in the brain as a unitary function but through multiple functionally heterogenous regions that jointly integrate to constitute the so-called attention network [25]. The fMRI study found that the frontoparietal network and the default mode network both play a critical role in sustaining attention tasks [26], and the prefrontal cortex simultaneously belongs to the two networks. Recently, some fNIRS studies also found that the prefrontal cortex was sensitive to different sustaining attention tasks [27,28]. Our fNIRS system cannot cover the whole brain like fMRI. We thus chose the prefrontal cortex, which is within the attention-related networks, as the training area. The channel location layout is shown in Figure 1.

### 2.3. Experiment Design

#### 2.3.1. Neurofeedback Design

**Figure 2:**
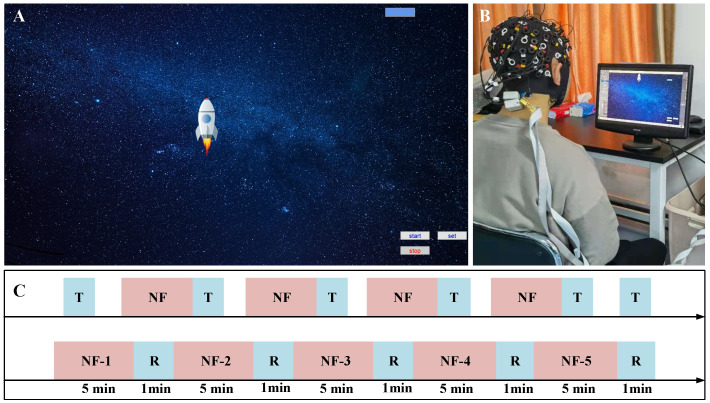
(**A**) neurofeedback system interface, (**B**) participant neurofeedback training scene, (**C**) training paradigm design; NF = Neurofeedback, T = Test, and R = Rest.

In this study, a self-developed python-based neurofeedback system was used to train participants’ attention levels. In order to make this system arouse higher levels of interest and avoid boredom caused by long-term training, a cartoon game was adopted. The system’s neurofeedback interface is shown in Figure 2A. Before training began, a pre-experiment was performed on participants to determine the training threshold and compare the Oxy-Hb concentrations in the task state to the threshold obtained in the pre-experiment. The cartoon game featured a rocket wherein if the Oxy-Hb concentration was higher than the threshold, the rocket would rise; otherwise, the rocket would fall.

The participants wore NIRSport electrode caps. The light source and detector were positioned in the forehead area, and the collected Oxy-Hb concentration signal was transmitted to a real-time data preset system through the local area network and the lab’s streaming layer data stream. A real-time data preprocessing module performed filtering preprocessing on the Oxy-Hb concentration data to remove motion artifacts and abnormal signals from the original signal. After the system processed the concentration signal, the neurofeedback information was calculated and fed back to participants in visual form. The participants adjusted themselves according to the visual neurofeedback information received and continued the training task, thereby forming a closed-loop neurofeedback training system.

For the INT group, the neurofeedback channel and threshold were individualized. The activation level of each channel of each participant was calculated. For each participant, the four channels with the highest activation level were used as the neurofeedback channels, and the averaged activation level of the four channels was used as the neurofeedback threshold. For the NINT group, the neurofeedback channel and threshold were determinated at the group level. The activation level of each channel was averaged within the group. The four channels with the highest activation level were selected as the neurofeedback channel, and the averaged activation level of these four channels was calculated as the neurofeedback threshold.

Before the start of the neurofeedback task, participants were told only to participate in a rocket lift-off game, but not given any strategy for raising the rocket. Individuals had to try all possible methods to raise the rocket. If the strategy adopted did not achieve a positive effect after 20 to 30 s, they could choose to change their strategy.

The study was conducted in a separate room, and participants performed their tasks on a computer. The training scene is shown in Figure 2B and the training paradigm in Figure 2C. Each participant performed a total of six runs of test tasks and four runs of neurofeedback. Each neurofeedback included five rounds of neurofeedback tasks. Each round of tasks lasted for five minutes. There was a one-minute rest period; each run of neurofeedback lasted for 30 min. The participants conducted a pre-test experiment in the first week. The test used a combination of improved AX version of continuous performance task (AX-CPT) and attention network test (ANT) paradigms. The neurofeedback channel and threshold were determinated in the AX-CPT pre-test experiment. In the second to fifth weeks, participants performed a neurofeedback task once a week. After each run of neurofeedback, the same test experiment as the previous was performed. Participants performed a post-test experiment in the sixth week; the experiment procedure was the same as the pre-test.

#### 2.3.2. AX-CPT Protocol

**Figure 3:**
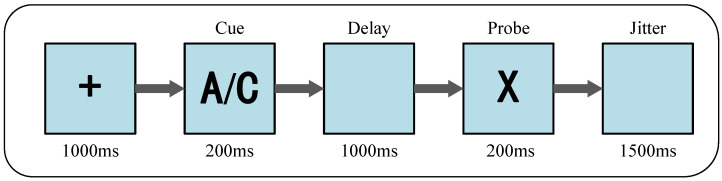
Task parameters and timing for the AX-CPT.

AX-CPT can be used to assess alertness and response inhibition. In the present research, the main process for this task is shown in Figure 3. A random stimulus was displayed in the center of the screen, either A, B, C, or D. It was present for 200 ms, and then there was a delay period of 1000 ms. Next, either X or Y was displayed randomly as a stimulus in the center of the screen. Participants were asked to give different button-based responses to the stimuli. In this experiment, the target stimulus was increased to two (e.g., AX became AX and CX), such that participants needed to be alert to the appearance of two stimuli at the same time, to a certain extent increasing the level of difficulty. In order to avoid the influence of habit on the results, AX-CPT was divided into 15 blocks. The target stimuli for each two adjacent blocks were different, prompting participants to always maintain a high level of attention and at the same time reducing the impact of the previous block on the next.

#### 2.3.3. ANT Protocol

ANT was designed to assess alertness, orientation, and conflict attention in a single test session [29]. Alertness refers to the ability to maintain a sensitive state and accept incoming information, orientation is the ability to select information from sensory input, and conflict refers to the ability to resolve conflicts through response.

**Figure 4:**
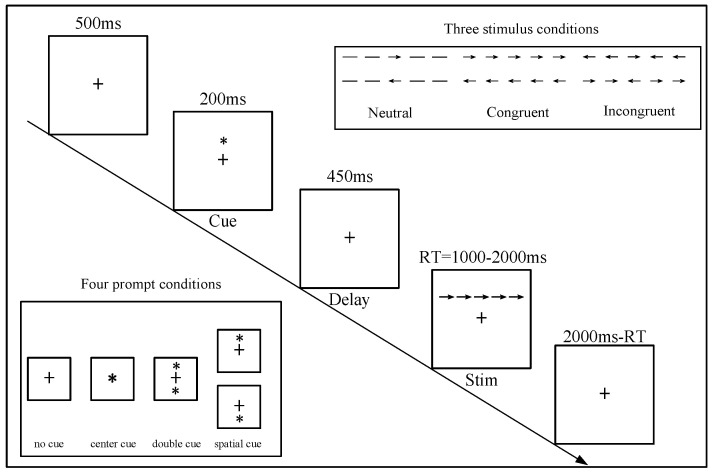
Task parameters and timing for the ANT.

The experiment process is shown in Figure 4. Before the start of each trial, the screen first showed a 500 ms fixation point “+” and then an alert signal for 200 ms. Next, a 450 ms fixation point appeared, and then the target stimulus was shown. If the target stimulus appeared to show no response within 2000 ms, participants entered the next trial. The average time for each trial was 3100 ms, and the program ran for 30 min. The formal experiment encompassed a total of 480 trials.

Alert signals were divided into four categories: no alert, central alert, double alert, and spatial alert. The four types of signals appeared randomly with the same level of probability. The target stimulus was five arrows. Participants needed to respond to the direction of the center arrow. The target stimulus was divided into three types: a congruent stimulus with five arrows in the same direction, incongruent stimulus with the central and surrounding arrows pointing in opposite directions, and neutral stimulus with only the arrow in the middle. The direction of the arrow and position (up or down) of the various stimuli were random, with the same level of task parameters and timing for the AX-CPT probability.

### 2.4. Data Analysis

#### 2.4.1. Data Analysis of Oxy-Hb during Neurofeedback

We extracted fNIRS data from four neurofeedback trainings, separately. Firstly, we filtered these data by a band-pass filtering (0.01–0.1 Hz) to remove the physiological noises, such as heartbeat (1 Hz) and respiration (0.4 Hz). In order to estimate the level of attention, we calculated the percentage of time that the Oxy-Hb concentration of each participant was higher than the threshold, and obtained the proportional change of each participant in the neurofeedback training. Then, we averaged the percentage within the group to compare the proportional change between the two groups.

We translated the level of sustained attention into the ability to continuously maintain Oxy-Hb concentrations above the threshold [30]. The length of time that the concentration of Oxy-Hb continued to exceed the threshold in each neurofeedback training was calculated. In each training, we selected the time period with a duration of more than 25 s for statistics to observe the change of sustained attention level.

#### 2.4.2. AX-CPT Data Analysis

To assemble statistics for the AX-CPT behavior data for all participants, we calculated the average reaction times and observed changes throughout the six tests. We then calculated the average reaction time and compared the average change trend of the two groups through the slope to verify whether different training modes will lead to different results. In order to test whether there was a significant difference in the reaction time between the two groups after training, we averaged the pre-test and post-test reaction time of the two groups, and then conducted a paired *t*-test.

We used the general linear model (GLM) to perform statistical analyses on the fNIRS data. The formula for the GLM was:

(1) y=xβ+e,

where *y* is the real fNIRS data of the AX-CPT task; *x* is the design matrix, which is the matrix generated by convolution of the boxcar of the AX-CPT task with the typical hemodynamic function; β is the GLM coefficient matrix, which represents the degree of similarity between the real data and design matrix (that is, whether there was significant activation in the real data during the task); and *e* is the residual. In the above formula, β is the unknown quantity we calculated. The β matrix represents the activation level. For each test of each participant, we calculated the corresponding β matrix.

#### 2.4.3. ANT Data Analysis

Three effect calculation methods were used for ANT. The alert network efficiency calculation was the non-alert signal average response time minus the central alert signal response time. The directional network efficiency calculation was the average of the upper and lower alert signal response times subtracted from the directional alert signal response time. The conflict network efficiency calculation was the average response time of the conflict stimuli minus the average response time of the consistent stimuli [29]. In order to test whether the effect is significant, we conducted a t-test on the three effects of ANT in each group.

We calculated the average reaction time of ANT in each of the two groups, then performed a paired t-test on the pre-test and post-test reaction times to estimate if the reaction time was improved significantly following training. Finally, we compared the trend of the average reaction time between the two groups.

## 3. Results

### 3.1. Changes of Brain Activity during Neurofeedback

#### 3.1.1. Proportion of Oxy-Hb Concentration above the Threshold

**Figure 5:**
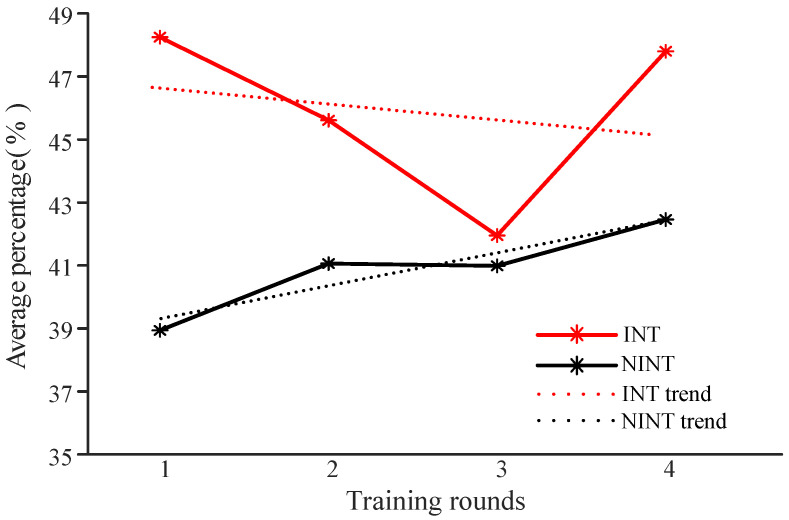
Proportion statistics for the Oxy-Hb concentration levels higher than the threshold resulting from the neurofeedback training.

The results of the INT group are shown in Figure 5 (in red). The overall trend for the INT group was downward for the first three times and upward for the third and fourth time. In the INT group, all participants showed an upward trend from the third to fourth training. The results of the NINT group are shown in Figure 5 (in black). The overall trend for the NINT group continued to increase. Compared with the first training, the proportion of the fourth training was greatly improved. The proportion of Oxy-Hb concentration above the threshold for the first three trainings of the INT group continued to decrease, but were still higher than for the NINT group.

#### 3.1.2. Duration of Oxy-Hb Concentration above the Threshold

**Figure 6:**
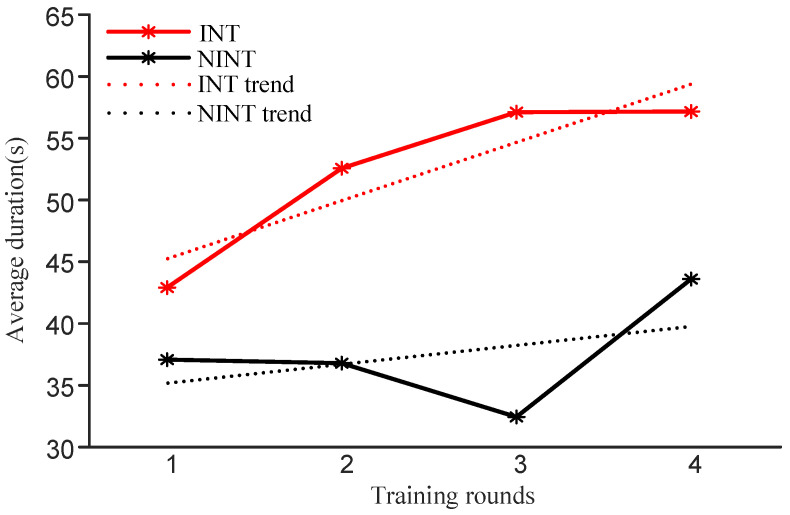
For both groups, the change in duration of Oxy-Hb concentration was higher than the threshold during the neurofeedback training.

The results of the INT group are shown in Figure 6 (in red). It can be seen that the participants maintained a high Oxy-Hb concentration signal. The average length of time above the threshold continued to increase throughout the four training sessions, indicating that the ability of the individuals in the INT group to maintain continuous attention improved after neurofeedback training. The results of the NINT group are shown in Figure 6 (in black). The average length of time the participants kept their Oxy-Hb concentrations above the threshold had an overall upward trend, but there was no continuous increase across the four training sessions. This indicates that the ability of participants in the NINT group to maintain sustained attention improved with the increase in the runs of neurofeedback, but did not achieve the effect of continuous improvement. Although the average length of time participants had Oxy-Hb concentrations higher than the threshold showed an overall upward trend throughout the training for both groups, the duration of the INT group increased faster than that of the NINT group. The trend line slope for the INT group ($k_{INT}$ = 4.73) was greater than the trend line slope for the NINT group ($k_{NINT}$ = 1.52).

### 3.2. AX-CPT Results

#### 3.2.1. Reaction Time

**Figure 7:**
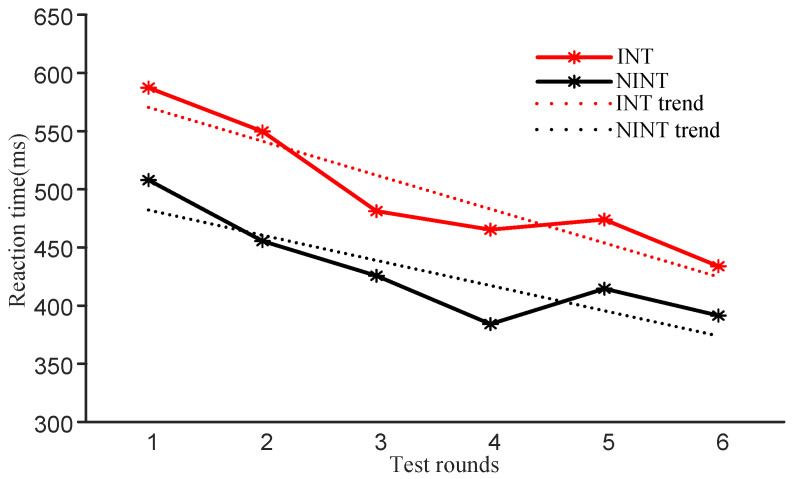
Changes in AX-CPT reaction times for the two groups.

The results of reaction time for the INT group are shown in Figure 7 (in red). The average reaction times for the INT group showed a downward trend, though it was not a continuous decrease. However, the overall decline was relatively large. Compared with the pre-test, the average reaction times for all participants in the INT group decreased significantly (p<0.05). The results of reaction time for the NINT group are shown in Figure 7 (in black). The average reaction time trend in this group decreased, but this was not a continuous effect (similar to the results for the INT group). The overall decline was relatively substantial, which is basically in line with the trends of participants in the INT group. The average reaction time of participants decreased significantly (p<0.05). The average reaction times for the INT and NINT groups both showed a downward trend. We calculated the linear trend for the two sets of data and found that the slope of the trend line for the INT group ($k_{INT}$ = −28.89) was greater than the slope of the trendline of the NINT group ($k_{NINT}$ = −21.33).

#### 3.2.2. Brain Activation

**Figure 8:**
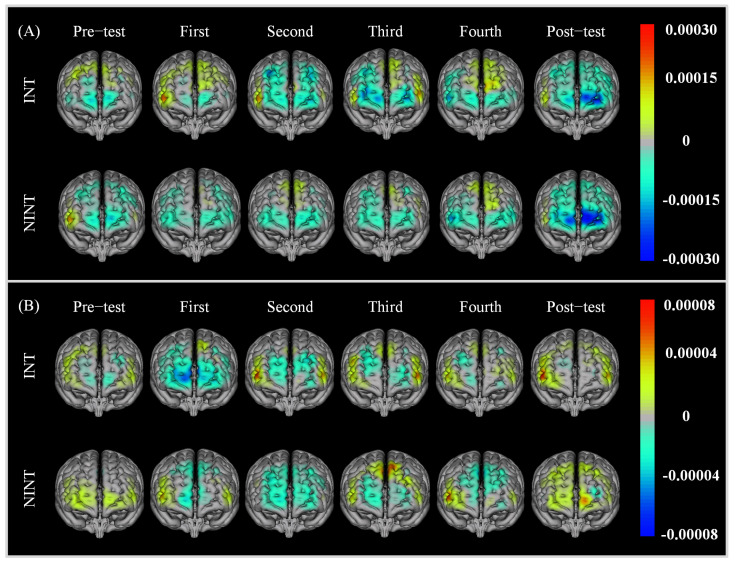
Average activation of (**A**) Oxy-Hb and (**B**) Deoxy-Hb for the two AX-CPT groups.

We used GLM to calculate the β matrix from the Oxy-Hb and Deoxy-Hb data for each group and obtained the brain activation of each test. The results are shown in Figure 8. For the Oxy-Hb data, The main activation areas of the INT and NINT groups were near the middle and dorsolateral prefrontal areas, which are relevant to the high-level cognitive functions such as short-term memory and auditory language attention. In the first five tests, for both groups, the participants’ activation areas gradually expanded. This was more obvious for the INT group. Compared with the NINT group, the INT group had more channels with positive activation in the first five tests. In terms of the average activation level, the INT group was also significantly higher than the NINT group (p<0.05). However, the results of the sixth test showed that the activation area became smaller and the position transferred to the right prefrontal cortex. For the Deoxy-Hb data, the activation of the INT group was mainly located at the dorsolateral prefrontal cortex, but that of the NINT group was mainly located at the middle prefrontal cortex. Along with the training, the average activation level of both groups increased gradually. Similar to the Oxy-Hb, the average activation level of the INT group was significantly higher than the NINT group after training (p<0.05). Different form the Oxy-Hb, the average activation level of the post-test was higher than the pre-test in both groups.

### 3.3. ANT Results

#### 3.3.1. Three Attention Effects

The *t*-test results of three effects in the INT group are shown in Table 1. The alertness, orientation, and conflict effects of participants in the INT group were significant for almost all of the ANT tests. Only in the first did the alertness effect not reach a level of significance. The *t*-test results of three effects in the NINT group are shown in Table 2. The orientation and conflict effects were significant for most of the ANT tests, but the alert effect did not reach significance in most.

#### 3.3.2. Reaction Time

**Figure 9:**
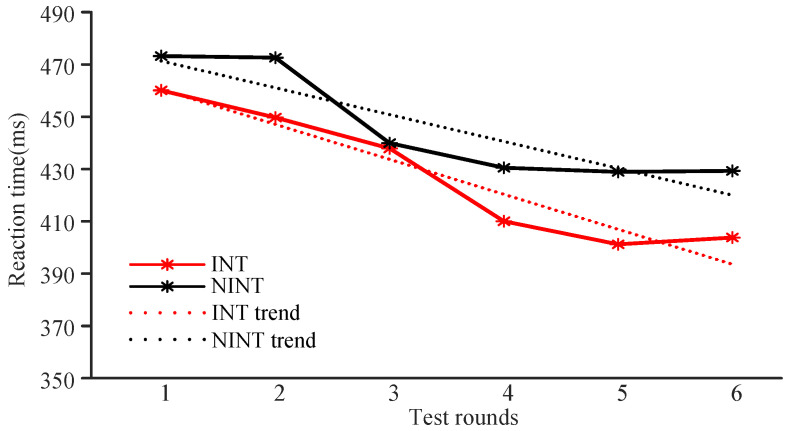
Changes of ANT reaction time in two groups.

Figure 9 (red) shows the change of ANT mean reaction time in the INT group. The average reaction time of the INT group showed a downward trend, but did not achieve the effect of continuous decline. In general, the ANT reaction time in the post-test of the INT group was significantly shorter than that in the pre-test (p<0.05). The results of the NINT group are shown in Figure 9 (black). The average reaction time of the NINT group also decreased, but it did not continue to decrease, which was similar to that of the INT group. The reaction time of post-test in the NINT group was significantly shorter than that of pre-test (p<0.05). In the last few tests, the average reaction time of the two groups showed a gradually stable trend. It can be seen that the post-test difference of reaction time between the two groups is higher than the pre-test, indicating that the reaction time of the INT group is reduced more. In the first five tests, the reaction time of the INT Group continued to decrease, while in the NINT group, the reaction time stabilized in the fourth test, and the training effect of INT may be more lasting. Further comparing the differences between the two groups, it was found that the slope of the trend line in the INT group ($k_{INT}$ = −12.99) was greater than that in the NINT group ($k_{NINT}$ = −10.29), and the average reaction time in the INT group decreased faster than that in the NINT group.

## 4. Discussion

In this study, we proposed an INT method that successfully improved participants’ attention levels. We compared its effect to that of a NINT program to verify the feasibility and superiority of the INT method. INT fully takes into account individual differences among participants and calculates INT channels for each, making the training effect more obvious. Data on brain activity and behavior were statistically examined in this study. The results revealed that the INT group outperformed the NINT group.

The fraction of oxygenated hemoglobin concentration above the threshold in the INT group was higher than that in the NINT group throughout the neurofeedback process. The reason for this phenomenon could be that in the INT group, each participant’s neurofeedback channels and thresholds are measured separately, which is very appropriate for each participant, making it easier for participants to regulate the activation of relevant channels; in the NINT group, neurofeedback channels and thresholds are obtained by all participants in the group. These channels and thresholds do not correspond to the participants’ personalities, making it difficult for them to adjust.

Concerning the length of time that Oxy-Hb concentration continues to be higher than the threshold during training, the average length of time for participants in the NINT group showed an overall upward trend, but there was no continuous increase in the results similar to what was seen for the INT group across all four training sessions. This indicated that for the NINT group, the sustained attention level of participants increased as the runs of neurofeedback increased, but the effect of continuous improvement was not achieved. For the INT group, the average lengths for the four training sessions continually increased, with the longest duration of each participant increasing to varying degrees. The reason for this phenomenon was that participants changed their ability to regulate their brain activity. The sustained attention level of the participants in the INT group increased as the number of trainings increased, and compared with the NINT group, the sustained attention level of the INT group achieved a continuous increase.

The behavior results of AX-CPT and ANT were analyzed. In terms of reaction time, both the INT and NINT groups’ average reaction times were on the decline. The decrease of average reaction time indicates that the judgment time of participants to stimuli is shortened, which indicates that the participants’ attention level is improved and the brain can process more stimulus information in a shorter time. The constant decrease in reaction time demonstrates that as the number of neurofeedback runs increases, the participants’ attention level improves.

The statistical results of the inter-group comparison revealed that the reaction time of the INT group declined faster than that of the NINT group in the behavioral results of AX-CPT and ANT. This shows that the INT group’s brain processed sensory information faster than the NINT group’s. This demonstrates that the INT strategy we offer is more effective and efficient. The training effect of the INT group is better under the same training times. It is worth noting that the statistical findings of the two tests revealed that, regardless of their beginning performance, participants’ reaction times fell. To put it another way, after neurofeedback training, the participants’ attention level will improve, regardless of their state prior to training.

Regarding the brain activation results from the AX-CPT task, the main activation area for the two groups was near the dorsolateral prefrontal area, a result consistent with the area of interest in certain previous studies [20]. The prefrontal lobe is an important functional area for various advanced mental activities such as attention and memory. The prefrontal cortex plays an important role in the selective attention process, and people with dorsolateral prefrontal damage have obvious imbalances when performing attention tasks [31]. In this study, the positive activation areas of the two groups spread gradually in the dorsolateral prefrontal lobe area, showing that neurofeedback training had a positive effect on brain activity and enhanced the activation degree of related training areas. Furthermore, the activation area of the INT group was much bigger than that of the NINT group, as was the activation intensity. The following are the key reasons behind this situation: One reason could be that participants in the INT group had a larger sensation of control over rockets during neurofeedback training, allowing them to receive more positive neurofeedback on a psychological level, resulting in increased motivation for brain regulation. Another reason could be that the INT group’s neurofeedback channel and threshold are better matched to the participants’ needs, making it easier for them to control brain activity. Because the common channel and threshold may not be adequate for some NINT group participants, the NINT group’s brain activity control impact is not as good as the INT group’s.

In the sixth AX-CPT imaging results, activation of the dorsolateral prefrontal area disappeared for the two groups, but it is worth noting that the activation areas were concentrated in the right anterior area of the prefrontal lobe. Because the sixth test was performed one week after the last training and the second to fifth tests were performed immediately after the neurofeedback training, the activation of the dorsolateral prefrontal area disappeared, possibly because the neurofeedback training lasted only for a short time. Thus, it could not achieve an effect that lasted for a week. In the first test, there was also activation in the dorsolateral prefrontal area. This may have been due to the fact that the participants were more mentally nervous when initially exposed to this paradigm, leading to more obvious activation. In previous studies, the efficacy of rtfMRI neurofeedback was tested in the right lower prefrontal cortex through a randomized controlled experiment. Researchers argued that this area was key, and is often damaged in ADHD patients [32]. This conclusion is consistent with the results of our sixth test. The activation areas were consistent, meaning that there were also key areas of attention-related activation in the sixth test, most likely due to the previous neurofeedback training. In sum, in almost all of the results, the performance of the INT program was better than that of the NINT program. This was because the INT program fully took into account individual differences among participants, achieving the greatest degree of fit and consequently resulting in a better training effect.

In addition, we recorded the strategies adopted by the participants in the neurofeedback training. In the NINT group, the strategies included imagining they were playing a game, imagining that they were driving a fighter plane or fighting a fire, and recalling their excellent performance in basketball. Participants in the INT group used a variety of efficient tactics, including recalling fast-paced songs or movie plots, mental arithmetic, multiplication, and reciting old poems. The strategies adopted by the two groups had certain aspects in common. The things they imagined were focused on the real world. Additionally, most participants said that the more detailed their imagination was, the more obvious was the rocket’s rise. This was because more focused attention is required when imagining details. The two groups adopted the same type of strategy, but the training results of the INT program were better, once again proving the merits of the INT program.

## 5. Conclusions

In this pilot study, we proposed an individualized fNIRS neurofeedback training system. The experimental results preliminarily prove that the proposed INT method can effectively improve the attention level, and its overall performance is better than that of the NINT method. To solve the limitation of sample number, we will supplement samples to further verify our proposed method. We hope this pilot study could provide a new perspective for attention improvement.

## Figures and Tables

**Table 1:** *t*-test of ANT effects in INT group.

Efficiency	Pre-Test		First		Second		Third		Fourth		Post-Test	
	*t*	*p*	*t*	*p*	*t*	*p*	*t*	*p*	*t*	*p*	*t*	*p*
Alert	4.08	0.01	2.30	0.08	3.57	0.02	2.12	0.10	5.10	<0.01	3.64	0.02
Directional	4.23	0.01	3.00	0.04	4.73	<0.01	3.18	0.03	3.02	0.04	3.73	0.02
Conflict	13.13	<0.01	5.59	<0.01	3.06	0.04	7.52	<0.01	7.82	<0.01	3.39	0.03

**Table 2:** *t*-test of ANT effects in the NINT group.

Efficiency	Pre-Test		First		Second		Third		Fourth		Post-Test	
	*t*	*p*	*t*	*p*	*t*	*p*	*t*	*p*	*t*	*p*	*t*	*p*
Alert	1.54	0.20	1.69	0.17	0.81	0.46	3.25	0.03	1.53	0.20	4.40	0.01
Directional	10.07	<0.01	13.01	<0.01	18.50	<0.01	8.15	<0.01	7.56	<0.01	4.69	<0.01
Conflict	6.55	<0.01	18.79	<0.01	14.00	<0.01	5.13	<0.01	7.68	<0.01	2.66	0.06

## Data Availability

The data presented in this study are available on request from the corresponding author.
